# Multistage Attitude Determination Alignment for Velocity-Aided In-Motion Strapdown Inertial Navigation System with Different Velocity Models

**DOI:** 10.3390/s19030665

**Published:** 2019-02-06

**Authors:** Shutong Li, Yanbin Gao, Meng Liu

**Affiliations:** 1 College of Automation, Harbin Engineering University, Harbin 150001, China; lishutong@hrbeu.edu.cn (S.L.); gaoyanbin@hrbeu.edu.cn (Y.G.); 2 Tianjin Navigation Instrument Research Institute, Tianjin 300131, China

**Keywords:** inertial navigation system, initial alignment, in-motion alignment, attitude determination-based initial alignment (ADIA)

## Abstract

A novel multistage attitude determination alignment algorithm with different velocity models is proposed to implement the alignment process of in-motion attitude determination alignment (IMADA) aided by the ground velocity expressed in body frame ($V^{b}$) in this paper. Normally, The $V^{b}$-based IMADA is used to achieve the coarse alignment for strapdown inertial navigation system (SINS). The higher the coarse alignment accuracy, the better initial condition can be achieved to guarantee the performance of the subsequent fine alignment. Consider the influence of the principal model errors and the calculation errors on the alignment accuracy in traditional $V^{b}$-based IMADA, this paper deals with a novel alignment algorithm by integrating two different velocity-based IMADAs and the multiple repeated alignment processes. The power of this novel alignment algorithm lies in eliminating the principal model errors and decreasing the calculation errors. Then, the higher alignment accuracy is achieved. Simulations and vehicle experiment are performed to demonstrate the validity of the proposed algorithm.

## 1. Introduction

Strapdown Inertial Navigation System (SINS) are now being widely used for position, location and navigation in both military and civil fields [1,2]. Nevertheless, SINS with integral operation is a dead-reckoning navigation system [3,4]. So it is necessary to research the system initialization, namely initial alignment, which is the precondition to guarantee the performance of navigation operation. Since the initial position and velocity can be easily obtained from the external aiding sensors, the core of initial alignment is to determine the attitude matrix between the body frame and the geographic frame [5]. Typically, this process consists of two stages: coarse alignment and fine alignment [6]. In the process of coarse alignment, misalignment angles are roughly obtained. Then, the precise misalignment angles are acquired from the subsequent fine alignment. Among many currently fine alignment methods, such as Kalman-based alignment and the compass alignment, however, their performance relies heavily on the coarse alignment [7,8,9,10]. The accurate coarse alignment can provide a good initial condition to guarantee the performance of fine alignment significantly. As a result, an accurate coarse alignment would be very necessary. 

Traditionally, the coarse alignment is usually accomplished analytically from gyroscope/accelerometer measurements of inertial measurement unit (IMU) [11]. Due to low signal to noise ratio of gyroscope in non-static base, however, the analytic coarse alignment is generally applicable to static alignment [12]. As a result, the attitude determination-based initial alignment (ADIA) method has been proposed for non-static coarse alignment [13,14]. With the matrix decomposition, the ADIA method has the characteristic of robustness to the external disturbance and vehicle maneuverability, thereby solving the problem of moving base alignment. Meanwhile, the process of initial alignment is also transformed into the solution of the known Wahba’s problem. This solution has also been studied for several decades and many methods of solution have been proposed [15,16]. For the solution of ADIA, two types of methods are usually employed, namely the vector cross product and the vector observations, such as ThRee-axIs Attitude Determination (TRIAD), Davenport’s q-method and so on [17,18,19]. In these methods, the vector observation based on the recursive Davenport’s q-method is preferable due to the superiority of quaternion and the adequate utilization of the vector information. Consequently, the ADIA assisted by the recursive Davenport’s q-method is applied to achieve the SINS initial alignment in this paper. 

On the other hand, the in-motion alignment has also been gain more attention recently and used in the special requirements, such as natural disaster and military applications [20,21]. For in-motion alignment, the external aided reference information must also be required. Alternatively, the external velocity information is usually used to aid the SINS in-motion alignment [22,23]. For the in-motion attitude determination alignment (IMADA), in presented works, the methods of velocity-aided IMADA can be divided into two categories: one is based on the ground velocity expressed in geographic frame ($V^{g}$) and another is based on the ground velocity expressed in body frame ($V^{b}$) [14,17,24]. Comparing with the one aided by $V^{b}$, the $V^{g}$-based IMADA has higher alignment accuracy and robust performance. And the aided velocity information relative to g-frame can be provided by external adding sensors, such as GPS. Nonetheless, $V^{g}$ must also be required correspondingly. Unfortunately, the GPS measurements are not always available, such as urban areas, underwater applications. In contrast to the $V^{g}$, whereas, the ground velocity information $V^{b}$ is easier to obtained from external navigation devices such as Odometers (OD), Doppler Velocity Logs (DVL) [25,26]. With the consideration of autonomy, moreover, the self-contained requirements (e.g., OD and DVL), instead of GPS, are strongly recommended to aid IMADA. As a result, the IMADA aided by $V^{b}$ is widely used to achieve the initial alignment [25,26,27,28]. 

Accordingly, some drawbacks such as slow convergence, vulnerability to divergence and low alignment accuracy have to be faced with $V^{b}$-based IMADA. In Reference [29], the backward process is proposed to improve the rapidity of IMADA, thus saving the alignment time. For the problem of anti-interference, the low-pass FIR filters is designed to eliminate the interferential acceleration [30]. In Reference [31], the infinite impulse response (IIR) low-pass filter is applied to attenuate the disturbance of the external aided velocity information. In Reference [32], the gyrocompass horizontal alignment algorithm is employed to attenuate the OD noises, thereby improving the robustness of the IMADA. For the third drawback of IMADA aided by $V^{b}$, one of the reasons is the noise errors of sensors, which is also the mainstream of research orientation [25,26,27,28,29,30,31,32]. However, few researches are presented for the two other reasons, namely, the calculation errors and the principled model errors. With IMADA aided by $V^{b}$, the ground velocity information relative to the geographic frame would not be provided directly and is usually obtained by the calculative attitude matrix and the $V^{b}$. As a result, the unavoidable calculation errors would degrade the attitude accuracy. In the implementation process, moreover, the omitted item of alignment model would result in the principle errors, thereby influencing on the alignment accuracy. In Reference [33], a Kalman-filtering-based IMADA for OD-aided SINS is designed to decrease the calculation errors. Besides, the dual-model ADIA algorithm is proposed to remove the principle errors, thereby improve the alignment accuracy [32]. 

Enlightened by the concept of the dual-model ADIA algorithm, a novel multistage attitude determination alignment algorithm with different velocity models is proposed to decrease the calculation errors and the principal model errors of the $V^{b}$-aided IMADA, further improve the alignment accuracy and can provide a good initial condition for subsequent fine alignment. The remainder of this paper is organized as follows. The main principles of IMADA aided by $V^{b}$ or $V^{g}$ are firstly presented in Section 2. The comparison and discussion of alignment results with two IMADA methods are also analyzed. In Section 3, the multistage attitude determination alignment algorithm with different velocity models is designed. Furthermore, simulations and experiments are carried out in Section 4 and Section 5, respectively. Finally, the conclusion is drawn in Section 6.

## 2. Attitude Determination Alignment for Velocity-Aided In-Motion SINS

In this paper, the initial frame is denoted by i, the Earth frame is denoted by e, the local geographic frame is denoted by g, the body frame is denoted by b. Furthermore, denoting the freezing inertial frames by g(0) and b(0), which are identical to the geographic frame and the body frame at time 0 respectively. Denoting the time-varying geographic frame and the time-varying body frame with time t by g(t) and b(t). Then, the attitude matrix $C_{b}^{g}(t)$, which is the heart of initial alignment, can always be decomposed into three matrix multiplication as follows.

(1) $$C_{b}^{g}(t)=C_{b(t)}^{g(t)}=C_{g(0)}^{g(t)}C_{b(0)}^{g(0)}C_{b(t)}^{b(0)}={\left(C_{g(t)}^{g(0)}\right)}^{T}C_{b}^{g}(0)C_{b(t)}^{b(0)}$$

where the matrix $C_{b}^{g}(0)$ is a constant matrix. Whereas, the attitude matrixes $C_{g(t)}^{g(0)}$ and $C_{b(t)}^{b(0)}$ are the time-varying matrixes. Since the coarse alignment is usually achieved in a short time, the changes of vehicle position can be ignored. As a result, the matrixes $C_{g(t)}^{g(0)}$ and $C_{b(t)}^{b(0)}$ can be calculated by

(2) $${\dot{C}}_{g(t)}^{g(0)}=C_{g(t)}^{g(0)}\left(\omega_{ig}^{g}\times \right)$$

(3) $${\dot{C}}_{b(t)}^{b(0)}=C_{b(t)}^{b(0)}\left(\omega_{ib}^{b}\times \right)$$

Where $\omega_{ig}^{g}=\omega_{ie}^{g}+\omega_{eg}^{g}$, $\omega_{ie}^{g}={\left[\begin{array}{ccc} 0 & \Omega \cos L_{0} & \Omega \sin L_{0} \end{array}\right]}^{T}$, $\omega_{eg}^{g}={\left[\begin{array}{ccc} -V_{N}/R & -V_{E}/R & -V_{E}\tan L_{0}/R \end{array}\right]}^{T}$, Ω is the angular rate of Earth’s rotation, $L_{0}$ denotes the initial latitude of vehicle, R represent the Earth radius, $V_{E}$ and $V_{N}$ denotes the East velocity and North velocity, respectively; $\omega_{ib}^{b}$ is obtained from the outputs of gyroscope; and $C_{g(t)}^{g(0)}(0)=I_{3}$, $C_{b(t)}^{b(0)}(0)=I_{3}$, $I_{3}$ denotes the identity matrix. 

With the above matrix decomposition, the acquisition of the attitude matrix $C_{b}^{g}(t)$ is transform into the determination of the constant matrix $C_{b}^{g}(0)$. Meanwhile, the matrix decomposition can also isolate the attitude changes by updating the matrixes $C_{g(t)}^{g(0)}$ and $C_{b(t)}^{b(0)}$ and can decrease the influence of the vehicle movement (including line movement and angular movement) on initial alignment, thereby achieving the SINS dynamic alignment. 

### 2.1. The Principle of Attitude Determination Initial Alignment for SINS

In ADIA, the initial constant matrix $C_{b}^{g}(0)$ is acquired by observing the gravitational apparent motion. With the constructions of the gravity vector observations and the observation equation, meanwhile, $C_{b}^{g}(0)$ can be determined by solving the well-known Wahba’s problem.

According to the relationship of coordinate transformation, we obviously have

(4) $$f^{g}=C_{b}^{g}f^{b}$$

Substituting Equation (1) into (4) and multiplying the left by $C_{g(t)}^{g(0)}$, we can obtain

(5) $$C_{g(t)}^{g(0)}f^{g}=C_{b}^{g}(0)C_{b(t)}^{b(0)}f^{b}$$

Defining the gravity observation vectors as follow

(6) $$\alpha (t)=C_{b(t)}^{b(0)}f^{b}$$

(7) $$\beta (t)=C_{g(t)}^{g(0)}f^{g}$$

According to Equation (5), the observation equation can be constructed as

(8) $$C_{b}^{g}(0)\alpha (t)=\beta (t)$$

With the Equation (8), the fundamental equation of attitude determination is constructed successfully. Further, the determination of the constant attitude matrix $C_{b}^{g}(0)$ is also the solution of the well-known Wahba’s problem. For the Wahba’s problem, many algorithms have been proposed. Here, only the recursive Davenport’s q-method is given and applied in this paper. The initial constant attitude matrix $C_{b}^{g}(0)$ can be written as the quaternion $q_{b}^{g}(0)$. Then the quaternion $q_{b}^{g}(0)$ can be obtained by the eigenvector associated with the largest positive eigenvalue of the four-dimensional matrix $K_{M}$, which is as follow.

(9) $$K_{M}=\left[\begin{array}{cc} B+B^{T}-\mathrm{tr}(B)I_{3} & \sum_{i=0}^{M}\beta_{i}\times \alpha_{i}\\ {\left(\sum_{i=0}^{M}\beta_{i}\times \alpha_{i}\right)}^{T} & \mathrm{tr}(B) \end{array}\right]$$

(10) $$B=\sum_{i=0}^{M}\beta_{i}\;\alpha_{i}^{T}$$

Where $\alpha_{i}$ and $\beta_{i}$ is the discrete time forms of the Equations (6) and (7). Further, the matrix $K_{M}$ can be calculated recursively by the following discrete equation.

(11) $$K_{k}=K_{k-1}+\left[\begin{array}{cc} \delta B_{k}+\delta B_{k}{}^{T}-\mathrm{tr}(\delta B_{k})I_{3} & \beta_{k}\times \alpha_{k}\\ {\left(\beta_{k}\times \alpha_{k}\right)}^{T} & \mathrm{tr}(\delta B_{k}) \end{array}\right]$$

(12) $$\delta B_{k}=\beta_{k}\alpha_{k}^{T}$$

where the initialization of matrix $K_{M}$ is set to zero matrix.

**Figure 1:**
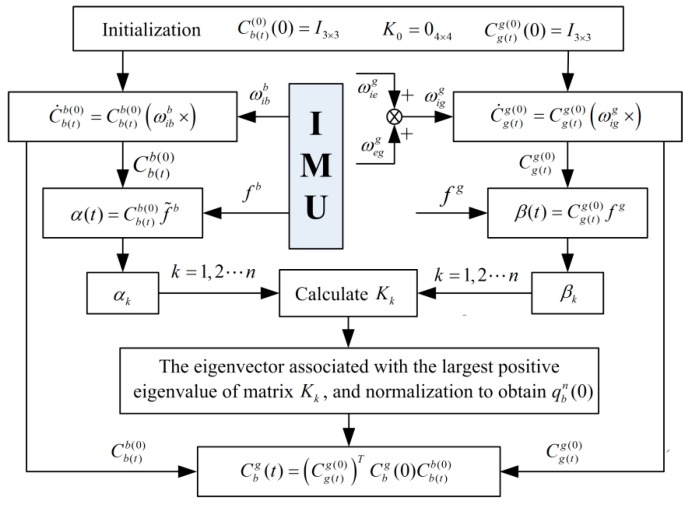
Implement process of the ADIA assisted by the recursive Davenport’s q-method.

According to above description, the ADIA assisted by the recursive Davenport’s q-method can be implemented as shown in Figure 1. 

### 2.2. The In-Motion Attitude Determination Alignment aided by $V^{g}$

For velocity-aided IMADA, the velocity observation vectors should be constructed to achieve the attitude determination since only the external velocity information can be utilized in the implement process of initial alignment. With the velocity observation vectors, meanwhile, the smoothness of integration can also be utilized adequately to restrain the periodic noise and Gaussian while noise, thereby improving the performance of the initial alignment. Based on $V^{g}$, next, the construction process of both the velocity vector observations and the observation equation are presented.

With the force equation of SINS, we have

(13) $$f^{g}={\dot{V}}^{g}+(2\omega_{ie}^{g}+\omega_{eg}^{g})\times V^{g}-g^{g}$$

Substituting Equation (13) into (4), multiplying the left by $C_{g(t)}^{g(0)}$ and integrating both sides, we can obtain

(14) $$C_{b}^{g}(0)\int_{0}^{t}C_{b(t)}^{b(0)}f_{ib}^{b}dt=\int_{0}^{t}C_{g(t)}^{g(0)}({\dot{V}}^{g}+(2\omega_{ie}^{g}+\omega_{eg}^{g})\times V^{g}-g^{g})dt$$

From Equation (14), the velocity vector observations and the observation equation relative to $V^{g}$ can be constructed as follow

(15) $$\alpha_{v}^{g}=\int_{0}^{t}C_{b(t)}^{b(0)}f_{ib}^{b}dt$$

(16) $$\beta_{v}^{g}=\int_{0}^{t}C_{g(t)}^{g(0)}({\dot{V}}^{g}+(2\omega_{ie}^{g}+\omega_{eg}^{g})\times V^{g}-g^{g})dt=\int_{0}^{t}C_{g(t)}^{g(0)}{\dot{V}}^{g}dt+\int_{0}^{t}C_{g(t)}^{g(0)}(\omega_{ie}^{g}+\omega_{ig}^{g})\times V^{g}dt-\int_{0}^{t}C_{g(t)}^{g(0)}g^{g}dt$$

(17) $$C_{b}^{g}(0)\alpha_{v}^{g}=\beta_{v}^{g}$$

Since

(18) $$\int_{0}^{t}C_{g(t)}^{g(0)}{\dot{V}}^{g}={C_{g(t)}^{g(0)}V^{g}|}_{0}^{t}-\int_{0}^{t}C_{g(t)}^{g(0)}\omega_{ig}^{g}\times V^{g}dt=C_{g(t)}^{g(0)}V^{g}-V^{g}(0)-\int_{0}^{t}C_{g(t)}^{g(0)}\omega_{ig}^{g}\times V^{g}dt$$

Substituting Equation (18) into (16), the Equation (16) can be rewritten as

(19) $$\beta_{v}^{g}=C_{g(t)}^{g(0)}V^{g}-V^{g}(0)+\int_{0}^{t}C_{g(t)}^{g(0)}\omega_{ie}^{g}\times V^{g}dt-\int_{0}^{t}C_{g(t)}^{g(0)}g^{g}dt$$

Similarly to the implement process of ADIA in Section 2.1, the in-motion attitude determination alignment aided by $V^{g}$ can be achieved with the Equations (15), (19) and (17).

### 2.3. The In-Motion Attitude Determination Alignment Aided by $V^{b}$

For IMADA aided by $V^{b}$, it would be different with the one aided by $V^{g}$ since only the external velocity $V^{b}$ can obtained. From [34], the force equation of SINS can be transformed and reorganized as follow

(20) $$C_{b}^{g}\left[{\dot{V}}^{b}+(\omega_{ie}^{b}+\omega_{ib}^{b})\times V^{b}-f_{ib}^{b}\right]=g^{g}$$

Substituting Equation (1) into (20), multiplying the left by $C_{g(t)}^{g(0)}$ and integrating both sides, we can obtain

(21) $$C_{b}^{g}(0)\int_{0}^{t}C_{b(t)}^{b(0)}\left[{\dot{V}}^{b}+(\omega_{ie}^{b}+\omega_{ib}^{b})\times V^{b}-f_{ib}^{b}\right]dt=\int_{0}^{t}C_{g(t)}^{g(0)}g^{g}dt$$

Similarly to the IMADA aided by $V^{g}$, the velocity vector observations and the observation equation relative to $V^{b}$ can be constructed as follow

(22) $$\alpha_{v}^{b}=\int_{0}^{t}C_{b(t)}^{b(0)}\left[{\dot{V}}^{b}+(\omega_{ie}^{b}+\omega_{ib}^{b})\times V^{b}-f_{ib}^{b}\right]dt$$

(23) $$\beta_{v}^{b}=\int_{0}^{t}C_{g(t)}^{g(0)}g^{g}dt$$

(24) $$C_{b}^{g}(0)\alpha_{v}^{b}=\beta_{v}^{b}$$

Since

(25) $$\int_{0}^{t}C_{b(t)}^{b(0)}{\dot{V}}^{b}dt=C_{b(t)}^{b(0)}{V^{b}|}_{0}^{t}-\int_{0}^{t}C_{b(t)}^{b(0)}\omega_{ib}^{b}\times V^{b}dt=C_{b(t)}^{b(0)}V^{b}(t)-V^{b}(0)-\int_{0}^{t}C_{b(t)}^{b(0)}\omega_{ib}^{b}\times V^{b}dt$$

Substituting Equation (25) into (22), the Equation (22) can be rewritten as

(26) $$\alpha_{v}^{b}=C_{b(t)}^{b(0)}V^{b}(t)-V^{b}(0)+\int_{0}^{t}C_{b(t)}^{b(0)}\left(\omega_{ie}^{b}\times V^{b}\right)dt-\int_{0}^{t}C_{b(t)}^{b(0)}f^{b}dt$$

According to above description, the IMADA aided by $V^{b}$ can also be achieved with Equations (26), (23) and (24). In the implement process, however, the projection of the angular rate of Earth’s rotation relative to b-frame is usually difficult to obtain. Consequently, the item of $\omega_{ie}^{b}\times V^{b}$ is omitted when the velocity vector observation $\alpha_{v}^{b}$ is calculated. And the $\alpha_{v}^{b}$ is approximated as

(27) $$\alpha_{v}^{b}\approx C_{b(t)}^{b(0)}V^{b}(t)-V^{b}(0)-\int_{0}^{t}C_{b(t)}^{b(0)}f^{b}dt$$

From Equation (27), the principled model errors of IMADA aided by $V^{b}$ would arise from the omitted item of alignment model, thereby resulting in the lower accuracy of coarse alignment and even possibly influencing the performance of subsequent fine alignment. As a result, the principled model errors of IMADA aided by $V^{b}$ should be considered to eliminate.

When the external velocity $V^{b}$ is applied to the aided information of IMADA, moreover, the ground velocity information relative to the geographic frame is usually only obtained by

(28) $${\hat{V}}^{g}={\hat{C}}_{b}^{g}(t)V^{b}$$

Where ${\hat{C}}_{b}^{g}(t)$ is the calculated strapdown attitude matrix. Then, the rotation angular rate of geographic frame $\omega_{eg}^{g}$ (namely, transport rate) can be obtained. Further, the attitude matrixes $C_{g(t)}^{g(0)}$ can also be updated with Equation (2). However, the calculated accuracy of the matrix ${\hat{C}}_{b}^{g}(t)$ is usually worse in the process of coarse alignment. As a result, the unavoidable calculation errors would degrade the accuracy of attitude matrixes $C_{g(t)}^{g(0)}$ and hence influence the accuracy of coarse alignment. 

According to the analysis above, the $V^{b}$—aided IMADA would have to suffer from the principled model errors and the calculated errors as compared with the IMADA aided by $V^{g}$ and hence influence the alignment accuracy. Excepting for the noise errors of sensors, hence, this two error sources should also be removed to further improve the alignment accuracy of coarse alignment. Sequentially, a good initial condition for fine alignment can also be acquired.

### 2.4. Comparisons and Discussion

**Figure 2:**
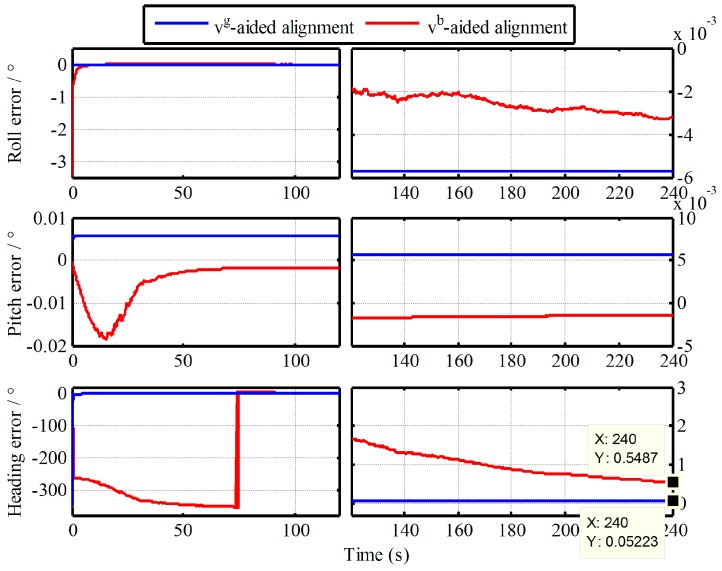
Comparison result of IMADA aided by two different velocity models.

In this section, the simulation comparisons of IMADA aided by $V^{b}$ and $V^{g}$ are performed. The simulation data are collected from the SINS simulator [35]. The main parameters of simulation are shown in Table 1. Moreover, the vehicle sails at the speed of 20m/s. Then, the external aided velocity information ${\tilde{V}}^{b}$ can be generated and a Gaussian white noise of standard deviation 0.03 m/s is also added intentionally as follow

(29) $${\tilde{V}}^{b}=\left[\begin{array}{c} 0\\ V^{b}\\ 0 \end{array}\right]+\left[\begin{array}{c} 0\\ 0.03\cdot \varepsilon_{randn}^{b}\\ 0 \end{array}\right]$$

where the $V^{b}$ is the vehicle speed, $\varepsilon_{randn}^{b}$ denotes the white noise. While the external aided velocity information $V^{g}$ is provided directly by the SINS simulator. Then, the alignment results of IMADA aided by $V^{b}$ and $V^{g}$ are shown in Figure 2. 

As shown in Figure 2, the IMADA aided by $V^{g}$ has obviously faster convergence speed and higher alignment accuracy as compared with the one aided by $V^{b}$. The convergence speed and the alignment accuracy of heading at 240s with two algorithms are 10s, 0.052° and 70s, 0.5487° respectively. As a result, this coincides with the analysis about the $V^{b}$-based IMADA, which have slow convergence speed, vulnerability to divergence and low alignment accuracy. This is because the $V^{b}$-based IMADA has to suffer from the principled model errors, the calculated errors, the noise errors of sensors and the indirectly acquired ground velocity information relative to g-frame.

**Figure 3:**
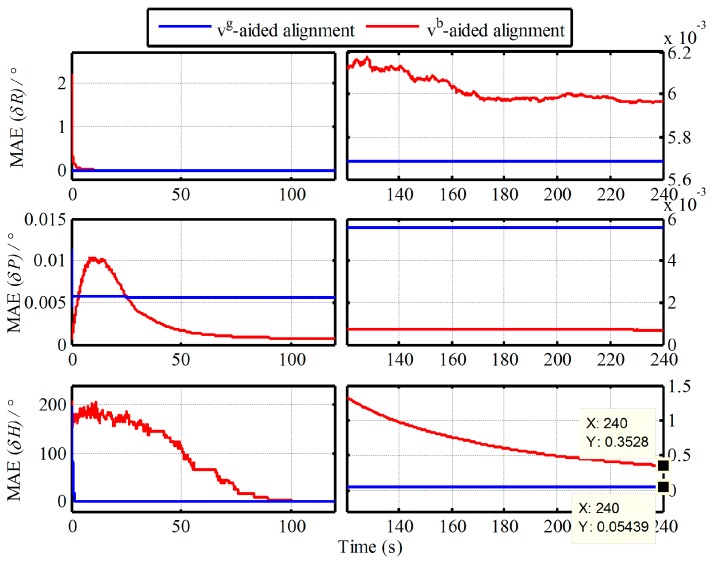
Curves of mean absolute deviation (MAE) of 50 alignment results.

**Figure 4:**
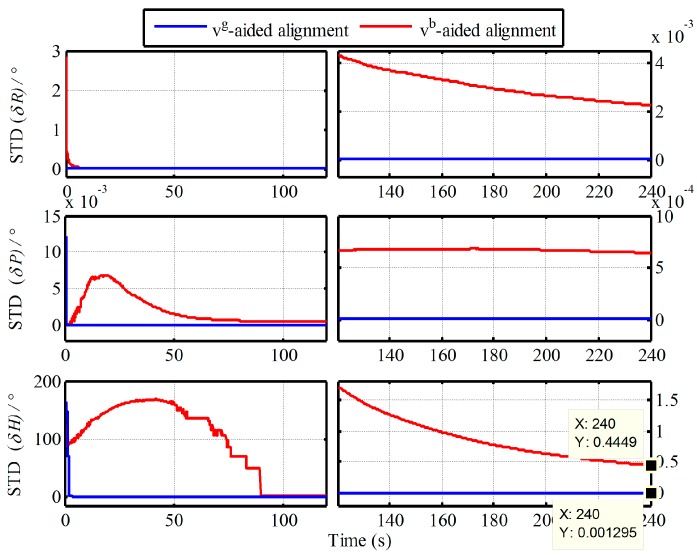
Curves of standard deviation (STD) of 50 alignment results.

Without loss of generality, moreover, the 50 simulation experiments with the Monte Carlo simulation are also conducted. The curves of mean absolute deviation (MAE) and standard deviation (STD) of 50 alignment results are shown in Figure 3 and Figure 4.

From Figure 3 and Figure 4, the MAE and STD of heading alignment with two algorithms at 240s are 0.3528°, 04449° and 0.05439°, 0.00129° respectively. The convergence speeds of heading based on $V^{b}$ and $V^{g}$ are 160 s and 10 s. As a result, the same conclusion is also obtained. When the aided information $V^{b}$ is selected as the external reference for in-motion alignment, the drawbacks from $V^{b}$-based IMADA need also to be faced. Consequently, those defections should be decreased to improve the alignment performance of IMADA, thereby guaranteeing the performance of the subsequent fine alignment significantly. 

## 3. The Multistage Attitude Determination Alignment with Different Velocity Models

As we have mentioned previously, the traditional $V^{b}$-based IMADA would suffer from the principled model errors, the calculated errors and the noise errors of sensors, thereby decreasing the coarse alignment accuracy and further influencing the subsequent fine alignment. As a result, those error sources should be removed. Fortunately, many studies have been presented to restrain the noise errors of sensors as described in Section 1. As a result, this paper would mainly focus on the suppression of both the principled model errors and the calculated errors.

**Figure 5:**
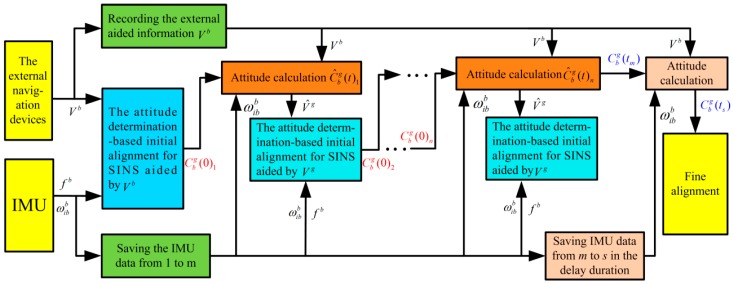
Block diagram of proposed multistage attitude determination alignment with different velocity models.

Notice that the $V^{g}$-based IMADA would never have the principled model errors. Consequently, the $V^{g}$-based IMADA algorithm can be applied to eliminate the model errors of the traditional $V^{b}$-based IMADA. Accordingly, the ground velocity relative to g-frame $V^{g}$ must also be required. With the external aided information $V^{b}$, however, only the $V^{b}$ can be obtained. As a result, the initial constant attitude matrix $C_{b}^{g}(0)$ should be firstly obtained with the traditional $V^{b}$-based IMADA. And then the required velocity information $V^{g}$ can be acquired by both the attitude matrix $C_{b}^{g}(0)$ and body-frame velocity $V^{b}$. Further, the implement problem of the $V^{g}$-based IMADA algorithm assisted can also be solved when only $V^{b}$ is provided as the external aided information. Nevertheless, the longer alignment time must also be required accordingly to execute the alignment process of $V^{g}$-based IMADA. This is contradictory with the rapidness requirement of initial alignment. With electronic technology development, fortunately, the capabilities of the navigation computer memory become large [8]. The navigation computer can save the IMU data and process them immediately. And hence the IMU data of SINS can be saved and be utilized repeatedly, thereby eliminating the above sacrifice for alignment rapidness. Moreover, the $V^{g}$-based IMADA can also be applied repeatedly to decrease the calculated errors. In this paper, therefore, a integrating with two different velocity-based IMADAs is applied to remove the model errors. And a multiple repeated alignment processes is proposed to decrease the calculated errors. The block diagram of the proposed multistage attitude determination alignment with different velocity models is also shown in Figure 5.

From Figure 5, the initial alignment is firstly carried out with the traditional $V^{b}$-based IMADA. Meanwhile, the IMU data and the external aided velocity information $V^{b}$ are also saved during the first initial alignment. With the saved data, the SINS data can also be utilized adequately without the sacrificingfor the alignment time. In the first alignment process, moreover, the initial constant attitude matrix $C_{b}^{g}{\left(0\right)}_{1}$ can also be obtained. With the matrix $C_{b}^{g}{\left(0\right)}_{1}$, then, the attitude matrix ${\hat{C}}_{b}^{g}{\left(t\right)}_{1}$ can be calculated accurately by Equation (1). According to the saved velocity information $V^{b}$, further, the ground velocity ${\hat{V}}^{g}$ can also be acquired with the Equation (28). And then the $V^{g}$-based IMADA would be would be applied as shown in Figure 5. As a result, the integrated IMADA algorithm with the two different velocity models can be utilized to eliminate the principled model errors. Only with one alignment process, nevertheless, the matrix $C_{b}^{g}{\left(0\right)}_{1}$ would still have the relatively larger errors. And the influence of the calculated errors of ${\hat{V}}^{g}$ on alignment performance would be still existed. Consequently, the multistage attitude determination alignment is proposed. Then, the $V^{g}$-based IMADA is carried out repeatedly to obtain the initial constant attitude matrixes $C_{b}^{g}{\left(0\right)}_{2}$, $C_{b}^{g}{\left(0\right)}_{3}$…$C_{b}^{g}{\left(0\right)}_{n}$ and improve accuracy of the initial constant attitude matrix gradually, thereby decreasing the influence of the calculated errors.

According to the above description, the multistage attitude determination alignment with different velocity models can remove the principled model errors and decrease the calculated errors, thereby improving the alignment performance of traditional $V^{b}$-based IMADA. On the other hand, suppose the time index of the IMU data is saved from m to s during the reciprocating delay process. Then the current attitude matrix $C_{b}^{g}(t_{s})$ can be also obtained by attitude calculation as shown in Figure 5. And then the process of fine alignment can also be carried out. Moreover, the procedure of the designed multistage IMADA method can also be summarized. Suppose the N-levels alignment process (N=2, 3, 4, ⋯) is applied, the implement steps are listed as follow.

**Step 1:** Initialization I. set k=0, n=1, $C_{g(t)}^{g(0)}(0)=I_{3}$, $C_{b(t)}^{b(0)}(0)=I_{3}$, $C_{b}^{g}(t)=I_{3}$, $K_{0}=0_{4\times 4}$.

**Step 2:**k=k+1, calculate the ground velocity $V^{g}$ according to Equation (28) and update $C_{g(t)}^{g(0)}$ and $C_{b(t)}^{b(0)}$ with the Equations (2) and (3).

**Step 3**: construct the $V^{b}$-based velocity vector observations $\alpha_{v}^{b}$ and $\beta_{v}^{b}$ according to the Equations (23) and (27).

**Step 4**: update the four-dimensional matrix $K_{k}$ with the Equations (11) and (12) according to the vector observations $\alpha_{v}^{b}$ and $\beta_{v}^{b}$ and calculate the eigenvector of the largest positive eigenvalue to determinate the initial attitude matrix $C_{b}^{g}(0)$.

**Step 5:** calculate the attitude matrix $C_{b}^{g}(t)$ according to Equation (1) and saving both the IMU data and the aided external body-frame velocity information $V^{b}$.

**Step 6**: go to step 2 until k=m.

**Step 7**: obtain the initial attitude matrix $C_{b}^{g}{\left(0\right)}_{n}=C_{b}^{g}(0)$.

**Step 8:** Initialization II. set k=0, $C_{g(t)}^{g(0)}(0)=I_{3}$, $C_{b(t)}^{b(0)}(0)=I_{3}$, $K_{0}=0_{4\times 4}$.

**Step 9:**k=k+1, calculate the attitude matrix $C_{b}^{g}(t)$ with the last obtained initial attitude matrix $C_{b}^{g}{\left(0\right)}_{n}$ according to Equation (1).

**Step 10:** calculate the ground velocity $V^{g}$ according to Equation (28) and update $C_{g(t)}^{g(0)}$ and $C_{b(t)}^{b(0)}$ with the Equations (2) and (3).

**Step 11:** construct the $V^{g}$-based velocity vector observations $\alpha_{v}^{g}$ and $\beta_{v}^{g}$ according to the Equations (15) and (19).

**Step 12:** update the four-dimensional matrix $K_{k}$ with the Equations (11) and (12) according to the vector observations $\alpha_{v}^{g}$ and $\beta_{v}^{g}$ and calculate the eigenvector of the largest positive eigenvalue to determinate the initial attitude matrix $C_{b}^{g}(0)$.

**Step 13:** go to step 9 until k=m.

**Step 14:**n=n+1 and obtain the initial attitude matrix $C_{b}^{g}{\left(0\right)}_{n}=C_{b}^{g}(0)$. 

**Step 15:** go to step 8 until n=N.

**Step 16:** obtain the attitude matrix at time m ($C_{b}^{g}(t_{m})$), $C_{b}^{g}(t_{m})=C_{b}^{g}(t)$.

**Step 17:** compensate the attitude variation during the delay according to the Equations (28), (2), (3) and (1); obtain the attitude matrix at current time $C_{b}^{g}(t_{s})$ to access the next fine alignment stage.

## 4. Simulations

**Figure 6:**
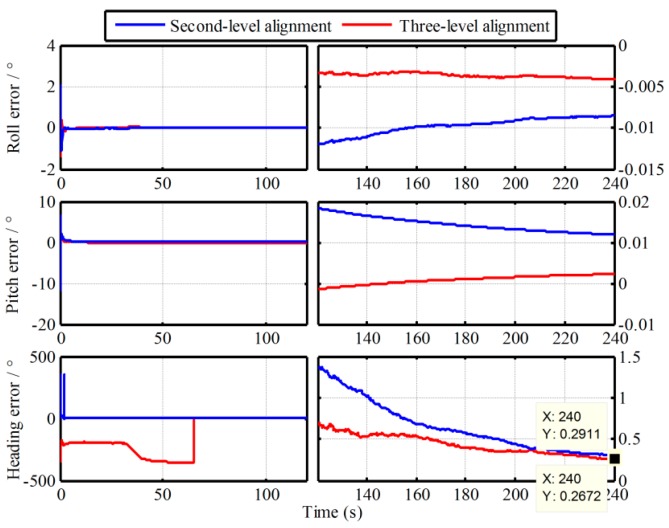
Errors curves of initial alignment with the proposed algorithm.

In order to verify the superior performance of the proposed algorithm, simulations is conducted in this section. With the same IMU data in Section 2.4, the proposed multistage attitude determination alignment is carried out with both the second-level alignment and three-level alignment. Namely, only the alignment algorithms of the $V^{b}$-based IMADA associated by one $V^{g}$-based IMADA or two $V^{g}$-based IMADAs are implemented. The alignment errors curves of three-axis are shown in Figure 6. 

From Figure 6, the alignment error curves with the proposed multistage attitude determination alignment algorithm are convergent with time. Comparing Figure 2 and Figure 6, moreover, the proposed algorithm has obviously higher alignment accuracy. The heading errors of the traditional $V^{b}$-based IMADA and proposed second-level alignment in 240s are 0.5487° and 0.2911°, respectively. This is because the principled model errors and the calculated errors are decreased with the proposed algorithm, thereby improving the alignment accuracy. Comparing the second-level alignment and three-level alignment in Figure 6, on the other hand, it is obvious that the three-level alignment would have higher alignment accuracy. The heading alignment accuracies of second-level and three-level alignments in 240s are 0.2911° and 0.2672°, respectively. This also coincides with the analysis above. Therefore, the proposed algorithm of multistage attitude determination alignment with different velocity models would have superior performance. With the multiple repeated alignment process of $V^{g}$-based IMADA, moreover, the calculated errors can be removed gradually. And we can notice that the improvement of alignment accuracy for traditional IMADA is decreased gradually with the repeated alignment process. The differences of the heading accuracy improvement with the two second-level alignment and three-level alignment are 0.2576° and 0.0239°. As a result, the influence of the calculated errors on the alignment performance would be decreased gradually until can be neglected. Thus, the proposed multistage IMADA can remove both the principled model errors and the calculated errors of traditional $V^{b}$-based IMADA, thereby improving the alignment accuracy.

**Figure 7:**
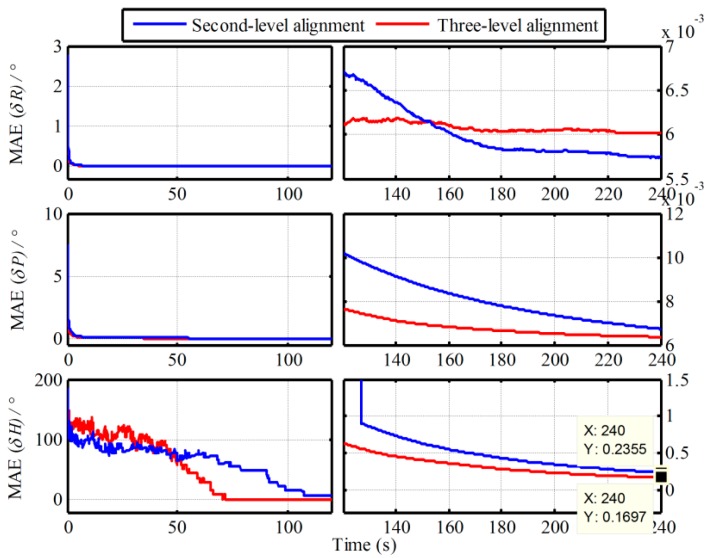
MAE curves of alignment results with the proposed algorithm.

**Figure 8:**
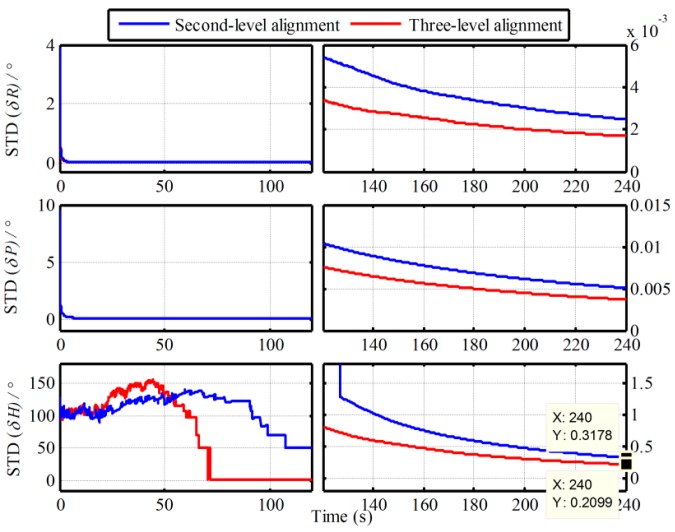
STD curves of alignment results with the proposed algorithm.

In addition, the Monte Carlo simulation with the same IMU data in Section 2.4 is also conducted. The MAE curves and the STD curves of 50 simulation experiments are shown in Figure 7 and Figure 8. The statistics of 50 heading alignment errors with the traditional and proposed algorithms in 240s are also shown in Table 2.

From Figure 7 and Figure 8, the error curves of MAE and STD are all convergent with time. Comparing with the MAE curves in Figure 3 and Figure 7, as well as the STD curves in Figure 4 and Figure 8, the convergent speeds of them cost almost the same time. The heading convergent speeds of the traditional $V^{b}$-based IMADA, the proposed second-level alignment and the proposed three-level alignment are 100s 105s and 70s. And the MAEs and STDs with the proposed algorithm are all lesser. The MAEs and STDs of heading errors in 240s are 0.3528°, 0.2355°, 0.1697° and 0.4449°, 0.3178°, 0.2099°. As result, the coincident conclusion above can also be obtained. The proposed algorithm can eliminate the principled model errors and the calculated errors of the traditional $V^{b}$-based IMADA, thereby improving the alignment performance. From Figure 7 and Figure 8, moreover, the three-level alignment would have higher alignment accuracy as compared with the second-level alignment. Consequently, the calculated errors can also be decreased gradually by the multiple repeated alignment process. From Table 2, on the other hand, the maximums of absolute value of heading errors with the four alignment processes are 0.0570°, 1.2229°, 1.0554° and 0.5713° respectively. As a result, the proposed $V^{b}$-based IMADA can improve the alignment accuracy of traditional $V^{b}$-based IMADA and has better statistic characteristics. However, it still has poor alignment performance relative to the $V^{g}$-based IMADA. This is mainly caused by the noise errors of sensors, such as the IMU and the external navigation devices. Therefore, the future efforts can focus on the restraint of the external noise to improve the alignment accuracy further.

## 5. Experiments

**Figure 9:**
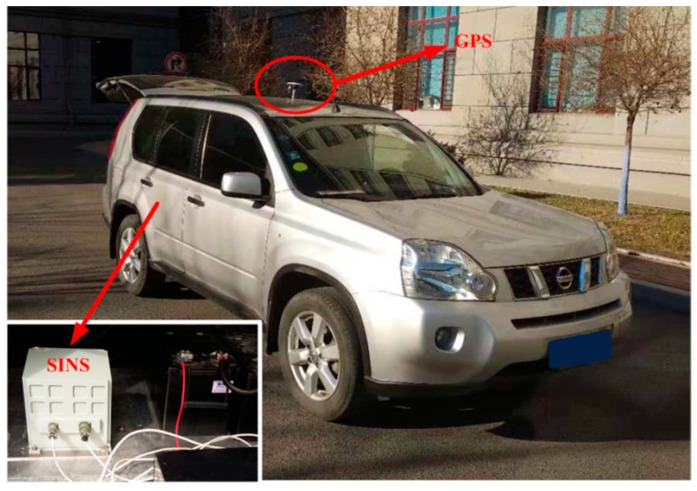
Vehicle experiment and equipment.

**Figure 10:**
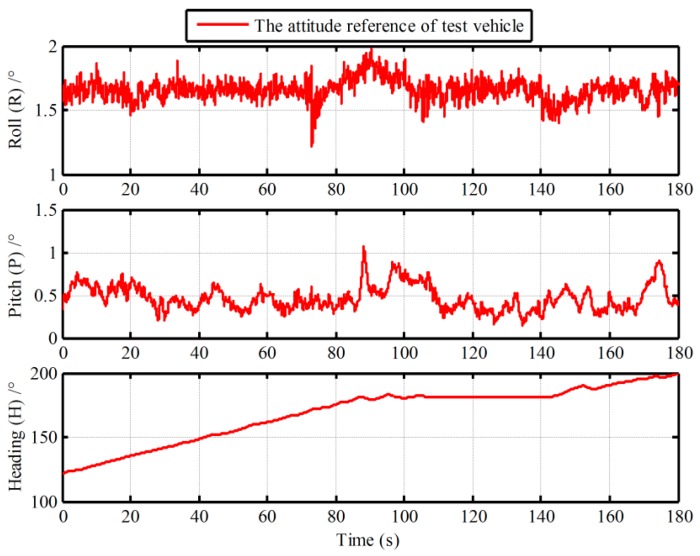
True attitude reference of vehicle experiment.

**Figure 11:**
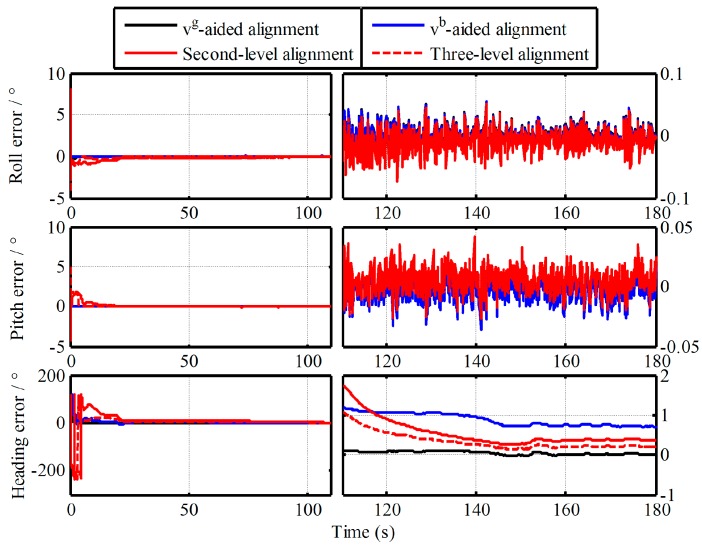
Alignment results of vehicle experiment.

In this section, the vehicle experiment was carried out to demonstrate the advantage of the proposed algorithm, as shown in Figure 9. In the vehicle experiment, a differential GPSs and a Fiber Optic Gyroscope (FOG) SINS are equipped. The gyro constant drifts and the accelerometer constant bias of the test FOG SINS are less 0.01 °/h and 100 µg, respectively. A 180s data segment of In-motion IMU is utilized. The local latitude and longitude of vehicle are 45.7778° and 126.6778°. In the process of initial alignment, the external aided velocity information $V^{g}$ is provided from the SINS/GPS integrated navigation system. While $V^{b}$ is obtained from the GPS. And the velocity error of GPS is 0.05 m/s (Root Mean Square—RMS). Meanwhile, the attitude outputs of the integrated navigation system are served as the reference of the IMADA coarse alignment. And the true attitude reference of vehicle experiment is shown in Figure 10. With the $V^{g}$-based IMADA, the traditional $V^{b}$-based IMADA, the proposed second-level alignment and the proposed three-level alignment respectively, the alignment results are shown in Figure 11. 

Form Figure 11, the curves of alignment errors with the four algorithms are all convergent with time. The alignment errors of heading in 180s are −0.0026°, 0.7095°, 0.3685°, 0.2008°. Obviously, the $V^{b}$-based IMADA with the proposed algorithm would have higher alignment accuracy as compared with the traditional one. As a result, the proposed $V^{b}$-based IMADA can remove the principled model errors and decrease the calculated errors, thereby improving the alignment accuracy. Moreover, it can also be seen that the three-level alignment would have higher alignment accuracy as compared with the second-level alignment. As a result, the proposed multistage attitude determination alignment would be feasible and favorable. The calculated errors can be decreased gradually by multiple repeated alignment process. Therefore, the proposed multistage attitude determination alignment algorithm with different velocity models would have superior performance and can improve the alignment accuracy of the traditional $V^{b}$-based IMADA, thereby guaranteeing a good initial condition for subsequent fine alignment further. Since the noises of aided velocity information $V^{b}$ from GPS measurements are always existed, however, the proposed algorithm still has a lower accuracy as comparing with the $V^{g}$-based IMADA from Figure 11. As a result, the external noise should be restrained to further improve the alignment accuracy in future. 

## 6. Conclusions

In this paper, the $V^{b}$-aided in-motion attitude determination alignment is investigated. Comparing with the $V^{g}$-based IMADA, the traditional $V^{b}$-based IMADA would have to suffer from the principal model errors and the calculation errors and hence owns lower alignment accuracy. A comparison experiment about the two IMADA methods is also presented to illustrate this phenomenon. Consequently, a novel multistage attitude determination alignment algorithm with different velocity models is proposed to implement the alignment process of $V^{b}$-based IMADA. The proposed algorithm combines with two different velocity-based IMADAs to eliminate the principal model errors and to decrease the calculation errors, improving the alignment accuracy. And the calculation errors can be decreased gradually in the multiple repeated alignment processes. As a result, the proposed $V^{b}$-based IMADA provides superior performance. Finally, the results of both simulations and vehicle experiment show that the proposed multistage attitude determination alignment algorithm can solve the designed drawbacks of traditional $V^{b}$-based IMADA and has higher alignment accuracy. 

## Figures and Tables

**Table 1:** Specifications of simulation parameters.

Parameters	Specifications
Initial position	Latitude: $L_{0}=45.6778^{{}^{\circ}}$, Longitude: $\lambda_{0}=126.6778^{{}^{\circ}}$
Initial attitude	Roll: 0°, Pitch: 0°, Heading: 30°
IMU	Gyroscope: constant drift 0.01 °/h, random noise 0.001 °/h
	Accelerometer: constant bias 100 μg, random noise 10 μg

**Table 2:** Statistics of the 50 heading alignment errors in 240s.

The Heading Errors in 240s	MAE (°)	Mean (°)	STD (°)	Maximum (°)	Minimum (°)	Max of Absolute Value (°)
$V^{g}$-based IMADA	0.0544	0.0544	0.0013	0.0570	0.0517	0.0570
$V^{b}$-based IMADA	0.3528	0.0076	0.4449	0.7273	−1.2229	1.2229
Second-level alignment	0.2355	0.0640	0.3178	1.0554	−0.4188	1.0554
Three-level alignment	0.1697	0.0160	0.2099	0.3376	−0.5713	0.5713
